# CANVAS: a late onset ataxia due to biallelic intronic AAGGG expansions

**DOI:** 10.1007/s00415-020-10183-0

**Published:** 2020-09-10

**Authors:** Natalia Dominik, Valentina Galassi Deforie, Andrea Cortese, Henry Houlden

**Affiliations:** 1 Department of Neuromuscular Disorders, UCL Institute of Neurology, Queen Square, London, WC1N 3BG UK; 2 Department of Brain and Behavioural Sciences, University of Pavia, Pavia, Italy

**Keywords:** Late-onset ataxia, CANVAS, *RFC1*, Repeat expansion, Southern blot

## Abstract

The ataxias are a group of disorders that manifest with balance, movement, speech and visual problems. They can arise due to dysfunction of the cerebellum, the vestibular system and/or the sensory neurons. Genetic defects are a common cause of chronic ataxia, particularly common are repeat expansions in this group of conditions. Co-occurrence of cerebellar ataxia with neuropathy and vestibular areflexia syndrome has been termed CANVAS. Although CANVAS is a rare syndrome, on discovery of biallelic expansions in the second intron of replication factor C subunit 1 (*RFC1*) gene, we and others have found the phenotype is broad and *RFC1* expansions are a common cause of late-onset progressive ataxia.

We aim to provide a review and update on recent developments in CANVAS and populations, where the disorder has been reported. We have also optimised a protocol for *RFC1* expansion screening which is described herein and expanded phenotype after analysing late-onset ataxia patients from around the world.

## Phenotype

**Fig. 1:**
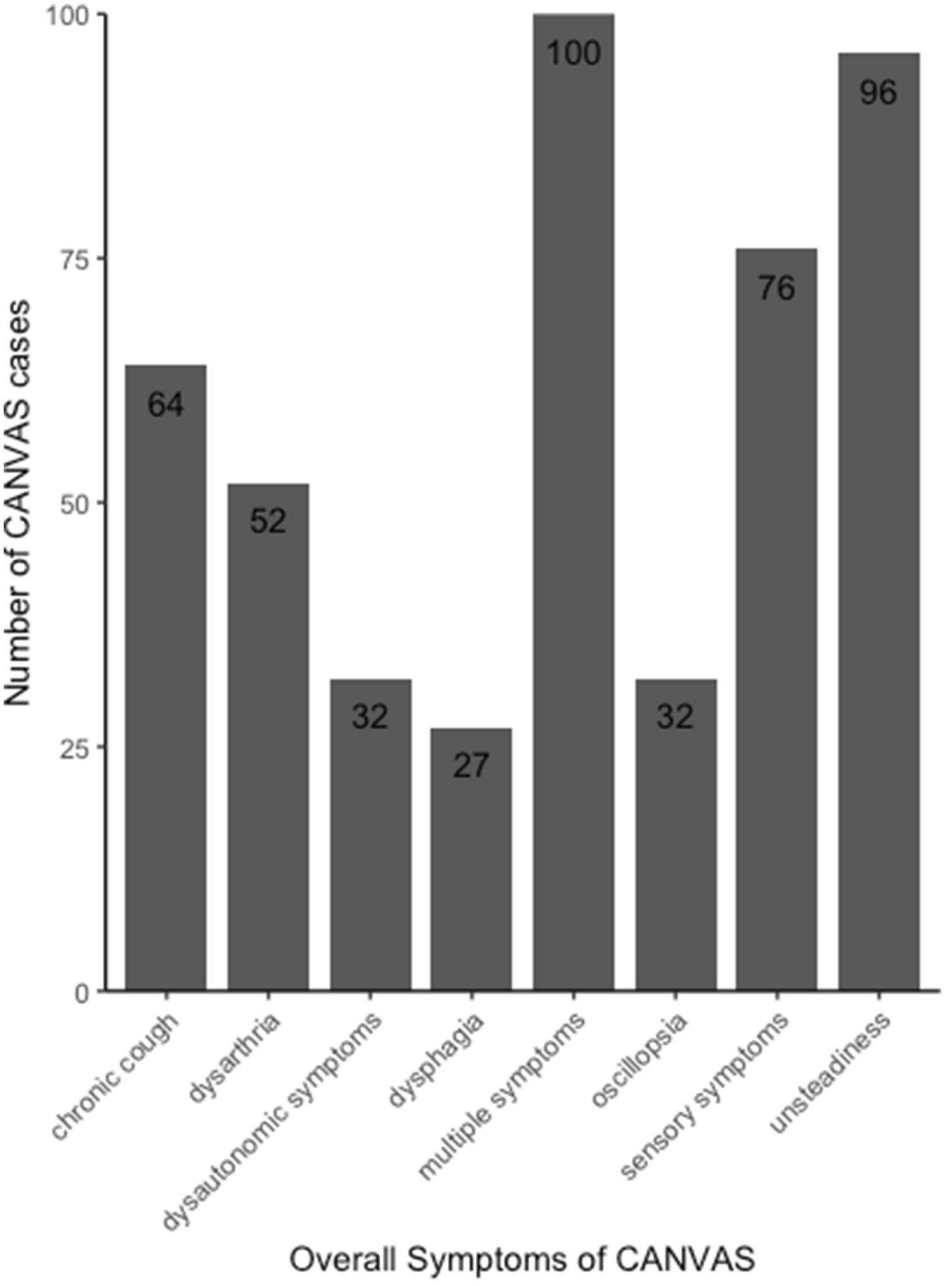
Overall symptoms of CANVAS. Symptoms during the manifestation of the disease in 100 biallelic *RFC1* expansion cases. Listed are the number of patients reporting specific symptoms and a combination of two or more symptoms (multiple symptoms) (Adapted from Cortese et al. 2020 [6])

Late-onset ataxia is a common neurological condition, where failure of systems controlling motor coordination occurs. This can lead to falls because of gait and stance ataxia and severe limitations in daily life. The disorder can be acquired, hereditary or non-hereditary; and up to 60% of familial and 19% of sporadic cases could have a genetic basis [1–3] and in most patients, it can present without an obvious familial background [4]. CANVAS is a common cause of late-onset progressive ataxia and the CANVAS patients suffer from ataxia, sensory neuronopathy or neuropathy as well as vestibular dysfunction [5]. Efforts have been made to piece together the syndromic clinical features of CANVAS with the genetic information to allow for more accurate clinical diagnosis. Recently, Cortese et al. reported the clinical features in the first 100 genetically confirmed *RFC1* CANVAS cases [6]. The mean age of onset appears to be just over 50 years. Progressive unsteadiness was the most common complaint at disease onset and universally present during disease progression. A sensory neuropathy was identified as a common feature in all cases carrying biallelic AAGGG *RFC1* expansions. There is often intrafamilial variability in age at onset and severity in clinical features but we have not seen extreme differences. The repeat sizing on Southern blot does not allow accurate expansion size comparison although as numbers of cases increase and our Southern blot method improves this may be possible in the future. Patients often reported symptoms including loss of feeling, neuropathic pain, ‘pins and needles’ (paraesthesia) and unpleasant sensation in response to touch (dysesthesia), pointing to a damage to peripheral nerves. Notably, in some patients the disease manifested as isolated sensory neuropathy. Cerebellar involvement was observed in two thirds of patients, showing nystagmus, dysmetric saccades and broken pursuits and leading, as the disease progresses, to dysarthria and dysphagia. A characteristic radiological pattern of cerebellar atrophy affecting the vermis and hemispheric crus I was identified and further confirmed on post-mortem brains [5]. Vestibular areflexia is also often present and probably its frequency is still underestimated. Patients may complain of oscillopsia and, when clinically tested, vestibulo-ocular reflex is often bilaterally impaired. Interestingly, over 60% of CANVAS patients experience dry cough (Fig. 1) whose cause remains unexplained. The cough is reported up to 30 years before neurological onset [6, 7], and it is hypothesised to be arising either as hypersensitivity syndrome due to a peripheral mechanism, where dysfunction of C fibres at level of upper way or oesophagus occurs; or due to cerebellar circuitry impairment [7]. Nerve conduction studies show non-length dependant sensory neuropathy in all the tested patients. Motor nerve conduction is preserved [8]. Visualisation of symptoms of 100 genetically confirmed CANVAS cases can be seen in Fig. 1 [6].

Moreover, Szmulewicz [9] and Cortese [6] both describe tests and assessments used for clinical diagnosis of patients. Briefly, nerve conduction studies are employed to check for sensory nerve involvement. Vestibular impairment should be tested by either of the following: bilaterally abnormal video head impulse test, bilaterally reduced caloric response or reduced vestibulo-ocular reflex gain tested using a rotatory chair. And finally, a range of tests should be employed in autonomic assessment, where parasympathetic and sympathetic functions should be checked. Abnormal blood pressure responses may be observed during change in posture and handgrip in sympathetic assessments. Parasympathetic function may be impaired which could be observed in heart rate variation during deep breathing or standing. Other autonomic nervous system dysfunctions may be present and can include affected micturition and defecation.

CANVAS shares common clinical features with other ataxias such as Friedreich Ataxia (FA) and Spinocerebellar ataxias (SCAs) and with Multiple System Atrophy (MSA) (Table 1). Therefore, clinical diagnosis of CANVAS may be difficult and genetic investigations should follow.

**Table 1:** Comparison of disorders with similar clinical manifestations to CANVAS

Disease	CANVAS	Friedreich ataxia	Spino-cerebellar ataxia	Multi system atrophy
**Gene**	RFC1	FXN	ATXN1-2-3, CACNA1, several others	No definite gene identified
**Cerebellar ataxia**	Frequent	Yes	Yes	Yes (MSA-C)
**Neuropathy**	Sensory neuropathy always present	Frequent sensory or sensory-motor neuropathy	Possible sensory or sensory-motor neuropathy depending on subtype	Usually absent
**Vestibular areflexia**	Frequent	Possible	Possible (SCA2)	Usually absent
**Dysautonomia**	Mild	Usually absent	Usually absent	Severe
**Onset**	Usually late onset	Usually early onset, but late onset possible	Usually early onset, but late onset possible	Usually late onset
**Additional neurological features**	Cough	Optic atrophy, hearing loss, pyramidal tracts involvement	Pyramidal tracts involvement, parkinsonism, cognitive impairment, visual impairment, variably associated depending on subtype	Parkinsonism, rapid progression, REM behaviour disorder
**Extra neurological involvement**	No	Cardiomyopathy, diabetes, scoliosis	No	No

CANVAS, FA, SCA and MSA patients share a number of complaints which may include ataxia, sensory neuropathy, dysarthria and dysphagia [5–9, 22–25]

### Repeat expansions in *RFC1*

CANVAS phenotype is especially complex, as is its underlying genetic cause. A healthy individual carries a nucleotide sequence of AAAAG in their second intronic region in the *RFC1* gene (hg19 chr4:39,350,045–39,350,103). The pentanucleotide is generally repeated 11 times; however, in affected individuals, the sequence is changed to AAGGG and the number of repeats becomes expanded from less than several hundred to more than 2000 times. The mutant repeat expansion is only pathogenic when present on both alleles. The disease can be either sporadic or occur in siblings. Notably, few families with cousins affected, suggesting a pseudo-dominant inheritance, were also reported; those individuals were biallelic for expanded AAGGG with one of the alleles coming from another branch of the family. The pathogenic AAGGG allele are fully penetrant, while so far, carriers of one AAGGG expanded allele are all unaffected [8]. Furthermore, biallelic (AAGGG)_exp_ appears to be CANVAS specific: no mutant configurations were found in two separate cohorts of patients, in a cohort of 336 multiple system atrophy mainly Caucasian patients from various brain banks and 102 Chinese Han MSA patients [10, 11].

Expansions of non-pathogenic repeat units including AAAAG, AAAGG, AAGAG and AGAGG were also observed [8, 12]. Thus far, the biallelic mutant AAGGG expansion is the only conformation shown to cause disease. Indeed, Cortese reported healthy individuals with conformations such as (AAAAG)_exp_/(AAGGG)_exp_, (AAAGG)_exp_/(AAGGG)_exp_ and (AAAAG)_exp_/(AAAGG)_exp_.

### Populations

The first cohort originating from 11 families with a CANVAS diagnosis studied by Cortese et al. consisted of 29 individuals of whom 23 were affected and six unaffected [8]. Additional cohort of 150 sporadic cases with late onset ataxia were screened and 22% of them were found to have the expanded AAGGG present and if only the individuals with sensory neuronopathy and/or bilateral vestibular areflexia were considered, the percentage would have been higher. The patients were of European ancestry [8]. Another cohort studied by Cortese included 363 Caucasian individuals with late-onset ataxia of whom 105 patients were identified to carry the biallelic (AAGGG)_exp_ [6].

In a bioinformatics-based approach to screening the repeat expansions, a cohort of 35 individuals with clinically diagnosed CANVAS was recruited. Of those, 30 were found to carry the mutant biallelic repeat expansion and most of the individuals were of European ancestry and a few were of different ethnic backgrounds [13].

There are currently not many studies on non-European cohorts. However, Akcimen et al. screened a cohort of Brazilian and Canadian patients with ataxia [12]. Out of total of 177 patients, they identified only one Brazilian family with two affected siblings that have the biallelic mutant expansion and one Canadian case. Interestingly, they report novel pentanucleotide sequences—AAGAG and AGAGG; however, the Repeat-primed polymerase chain reaction (RP-PCR) plot for AAGAG does not show a typical saw tooth pattern but they further explain that Sanger sequencing was needed for its identification.

A few CANVAS cases were identified in Japan [14–16], of which one was genetically confirmed by long read sequencing and it has the same haplotype around *RFC1* as most European cases [16].

Fan and colleagues screened a cohort of late-onset ataxia patients found in the Chinese Han population [11]. They established that the frequency of heterozygous AAGGG repeat expansion alleles in their ataxia cohort was similar to their multiple system atrophy cohort: 2.75% and 2.45%, respectively; however, no biallelic cases were found. Wu and colleagues described 26 CANVAS patients in New Zealand, all of them were of European ancestry with four patients also having New Zealand Maori and two patients having Polynesian ancestries [17].

The frequency of the (AAGGG)_exp_ allele differs between populations worldwide. The majority of the reported CANVAS cases are European and indeed an ancestral haplotype has been identified and it is estimated to have emerged more than 25,000 years ago, likely in Europe [13]. In a cohort of European descent, the allelic distribution for (AAGGG)_exp_ was concluded to be 0.7% and conversely, the wild type, non-expanded AAAAG allele frequency equals 75.5% [8]. In a Canadian cohort of 163 control individuals, the frequency of expanded AAGGG was 4% and non-expanded AAAAG, 84.6% [12]. In 490 healthy Chinese Han individuals, the frequency of (AAGGG)_exp_ was found to be 2.24% and for (AAAAG)_11_, 70.82% [11].

Based on allele frequency the estimated disease prevalence at birth ranges from 1:10,000 to 1:650 individuals [6]. Wu et al. estimated the disease prevalence in Auckland, New Zealand to be nearly 1:100,000 [17].

It appears that the AAGGG mutant expansion is a common cause of late onset-ataxia and it is underdiagnosed or misdiagnosed due to a range and variety of symptoms as well as relatively recent characterisation of the disorder [5, 18, 19] and identification of the underlying genetic defect [8]. The incidence of (AAGGG)_exp_ alleles is at its highest (92%) in cases with typical CANVAS symptoms, this decreases; however, when only cerebellar ataxia with sensory neuropathy are characterised [6]. It is, therefore, of great importance that more individuals from different backgrounds are screened for possible *RFC1* pentanucleotide expansions to better characterise the disorder and aid clinical diagnosis as well as estimation of the global prevalence of (AAGGG)_exp._

### Investigation methods for pentanucleotide expansions

RP-PCR and flanking PCR are performed as previously described by Cortese et al. on genomic DNA [8]. Flanking PCR is employed to amplify the intronic *RFC1* region and an affected individual will not show an amplified product on an agarose gel as opposed to non- affected individuals, where at least one PCR product corresponding to non-expanded AAAAG or intermediately expanded AAAAG, AAAGG or AAGGG can be amplified.

**Fig. 2:**
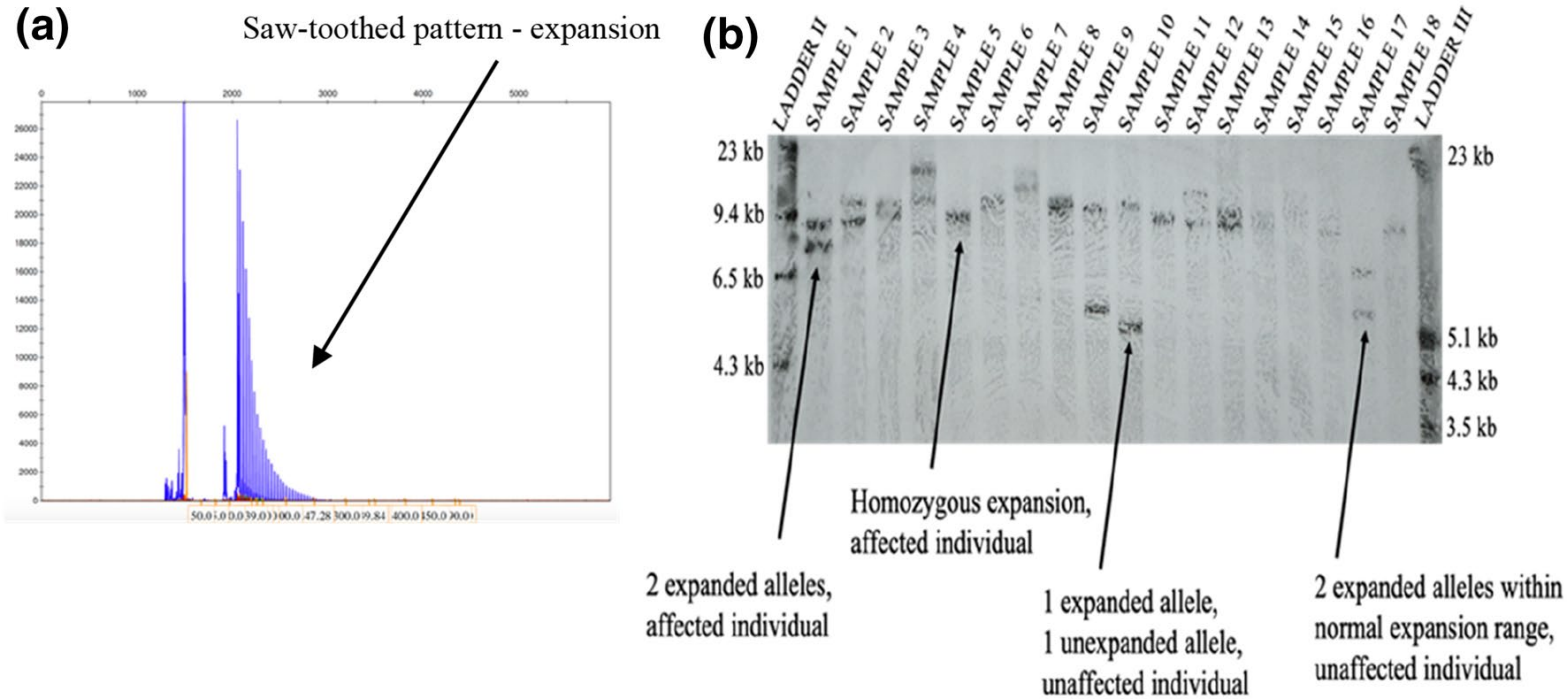
RP-PCR and Southern blotting. **a** RP-PCR with primers targeting the AAGGG pentanucleotide repeated unit. An ABI 3730 DNA Analyser was used to separate the products, and these were visualised using GeneMapper. The presence of a ‘sawtooth’ pattern is characteristic of a possible affected individual **b** Patients are characterised by either one overlapping band or two bands within the region 7–15 kb. Carriers are identified with one band residing between 7 and 15 kb and the other at 5–6.5 kb, equivalent to the non-expanded AAGGG sequence or a small AAGGG expansion. Non-affected individuals exhibit two bands in regions between 5 and 6.5 kb. Two ladders are needed for accurate measurements: DIG-labelled DNA Molecular Weight Marker II (Roche) (labelled as LADDER II) and DIG-labelled DNA Molecular Weight Marker III (Roche) (labelled as LADDER III). The left- and right-hand side of each panel documents the molecular weights represented by LADDER II and LADDER III, respectively. Overnight transfer with a 10 min exposure to the Fluorescent Detection Film

In patients, RP-PCR shows a typical saw-tooth decremental pattern (Fig. 2a) for AAGGG, but not AAAAG or AAAGG. Absence of amplified flanking PCR product together with saw-tooth decremental pattern on RP-PCR are highly predictive of biallelic *RFC1* expansion. It is important that flanking PCR and RP-PCR are used alongside and interpreted with caution. In rare instances, we observed no flanking PCR product in controls and negative RP-PCR for mutant expansions; however, positive RP-PCR for non-pathogenic expansions were found. Moreover, it is possible that flanking PCR will show no amplifiable product but positive RP-PCR for one allele with expanded AAGGG and one allele with expanded AAAGG for a non-affected individual.

**Fig. 3:**
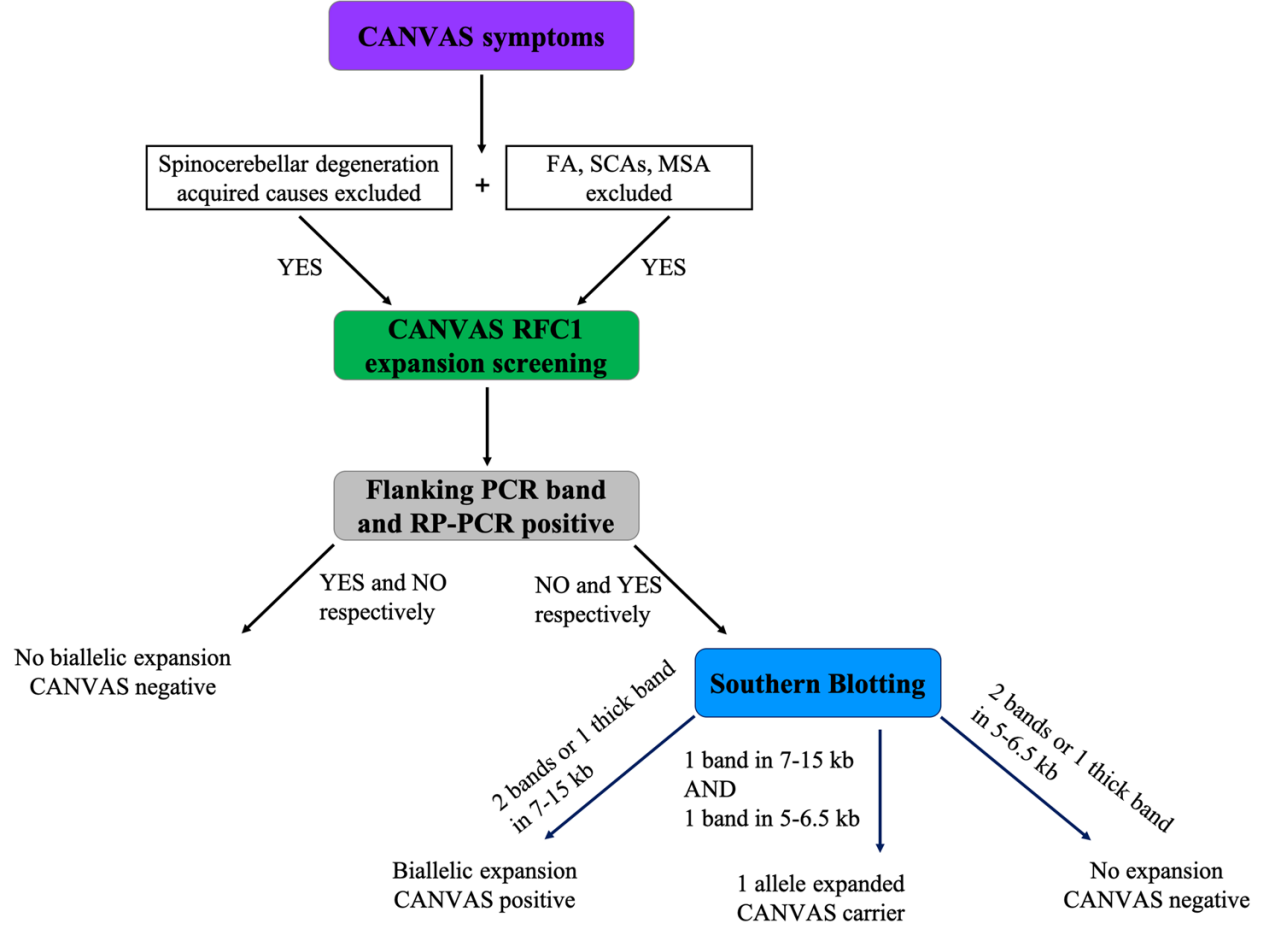
Work flow diagram representing repeat expansion screening methodology. Flanking PCR and RP-PCR are used simultaneously on patient DNA to identify which are more likely to have two expanded alleles. If flanking PCR shows no amplifiable product and RP-PCR shows typical saw-tooth pattern, southern blotting is carried out on additional patient DNA if available

Where cases likely positive for the biallelic AAGGG expansion in *RFC1* are found, provided sufficient DNA is present, Southern blotting should be used for further confirmation of PCR results. Southern blotting is the current gold standard in measuring repeat expansions [20]. The screening process for pentanucleotide expansions is shown in Fig. 3.

We have optimised the Southern blot protocol for *RFC1* screening (Fig. 2b). Briefly, the changes from the original protocol, which produced good images in a lot of cases, include an overnight transfer of DNA to a positively charged nylon membrane as opposed to a transfer lasting 4 h which in a few cases resulted in an incomplete DNA transfer to the membrane (not shown). After transfer, pre-hybridisation was extended to 5 h from the original 3 h and the temperature was increased to 49 °C from 46 °C. The increase of temperature was also implemented for the overnight hybridisation step. While five micrograms of good quality genomic DNA are still needed, bands are generally better visible and more blots are successful. Moreover, shorter exposure time for visualising the bands on fluorescent detection film can be used which leads to less background and higher quality image overall, we find that 10 min exposure produces clearest image. Biallelic expansions in affected individuals are seen as two bands between 7 and 15 kb, or one thicker band if the expansions on both alleles are the same or similar size. Unaffected individuals who carry the mutation can either have one band in normal, wild type, range of 5 kb and one band in expanded range of 7–15 kb, or two expanded alleles—one in the non-pathogenic range of up to around 6.5 kb and one in the pathogenic range. Although Southern blotting is greatly important in repeat expansion screening, it has its limitations. Often, there might not be DNA available to perform the blotting as relatively large quantity (5 µl or more) of good quality (260/280 ratio of 1.8–2 and 260/230 ratio of 2–2.2) DNA should be used. It is a time-consuming and labour intensive technique that requires a specific laboratory set-up and might fail at various stages; however, with our protocol optimisation, we observe good quality images.

Regardless of the limitations, we recommend that Southern blotting is used further to flanking PCR and RP-PCR in CANVAS screening.

Long-read sequencing can also be successfully employed to detect pathological repeat expansions as shown by Nakamura et al. [16]. Briefly, they obtained long reads from the patient and normal control DNA using PromethION sequencer (Oxford Nanopore Technologies, Oxford Science Park, UK). Then, they enriched the repeat regions with Cas9-mediated system and sequenced them using a MiniION nanopore sequencer. Human reference genome (hg38) was used to align the reads and tandem-genotypes v1.1.0 was used to conduce tandem repeat genotyping and multi-dataset prioritisation. Finally, *RFC1* repeat reads containing ± 50-bp flanking regions were aligned to generate a consensus repeat sequence.

### The disease-causing mechanism

Currently, the mechanisms causing CANVAS are unknown, and this is, therefore, an ongoing area of research. The recent discovery of the recessive AAGGG repeat expansion in the *RFC1* gene and has been identified as a frequent cause of this late-onset disease. This mutated expansion was compared to an expanded form of the reference sequence, AAAAG. Unlike the pathogenic AAGGG_exp_, individuals with either one or two copies of AAAAG_exp_ do not exhibit symptoms. The AAAAG_exp_ is considerably smaller than AAGGG_exp_, which indicates that both nucleotide change and expansion size have an impact on the pathogenic mechanism [8].

As the pathogenic repeat expansion is inherited in a recessive manner, it would be suspected that the mechanism of the disease would be a loss of function [21]. However, preliminary investigations mostly in non-affected tissues did not show a clear reduced expression of canonical *RFC1* transcript or protein [8]. However, this does not exclude other effects that this repeat expansion may have, for example altering the organisation of chromatin or effect on other transcripts or isoforms or tissue specific consequences. These uncertainties about the underlying mechanism may be more complex than previously hypothesised and further research is needed to elucidate the pathogenic events in CANVAS patients.

## Conclusions

Homozygous AAGGG expansions in *RFC1* gene appear to be a common cause of late onset ataxia. Efforts are being made to better characterise the phenotype of CANVAS disorder to aid clinical diagnosis. Most common complaints in patients with CANVAS include progressive unsteadiness at disease onset and during progression, sensory neuropathy and vestibular areflexia. In addition, cerebellar involvement leading to dysarthria and dysphagia may be observed. Patients may complain of oscillopsia and over 60% may experience unexplained dry cough. A variety of laboratory tests needs to be employed in parallel to find likely positive cases and those tests are cumbersome and relay on large quantities of genomic DNA. We improved Southern blotting which is a good technique for confirming the expansions; however, it has a variety of limitations and long read sequencing or other techniques could be sought out. The disease mechanisms are still elusive and further studies are needed to understand the pathogenicity of the mutant pentanucleotides.

## Data Availability

Created in our laboratory.

## References

[1] Muzaimi MB, Thomas J, Palmer-Smith S, Rosser L, Harper PS, Wiles CM, Ravine D, Robertson NP (2004). Population based study of late onset cerebellar ataxia in south east Wales. J Neurol Neurosurg Psychiatry.

[2] Gebus O, Montaut S, Monga B, Wirth T, Cheraud C, Alves do Rego C, Zinchenko I, Carre G, Hamdaoui M, Hautecloque G, Nguyen-Them L, Lannes B, Chanson JB, Lagha-Boukbiza O, Fleury MC, Devys D, Nicolas G, Rudolf G, Bereau M, Mallaret M, Renaud M, Acquaviva C, Koenig M, Koob M, Kremer S, Namer IJ, Cazeneuve C, Echaniz-Laguna A, Tranchant C, Anheim M (2017). Deciphering the causes of sporadic late-onset cerebellar ataxias: a prospective study with implications for diagnostic work. J Neurol.

[3] Lieto M, Roca A, Santorelli FM, Fico T, de Michele G, Bellofatto M, Sacca F, de Michele G, Filla A (2019). Degenerative and acquired sporadic adult onset ataxia. Neurol Sci.

[4] Klockgether T (2010). Sporadic ataxia with adult onset: classification and diagnostic criteria. Lancet Neurol.

[5] Szmulewicz DJ, Waterston JA, Halmagyi GM, Mossman S, Chancellor AM, McLean CA, Storey E (2011). Sensory neuropathy as part of the cerebellar ataxia neuropathy vestibular areflexia syndrome. Neurology.

[6] Cortese A, Tozza S, Yau WY, Rossi S, Beecroft SJ, Jaunmuktane Z, Dyer Z, Ravenscroft G, Lamont PJ, Mossman S, Chancellor A, Maisonobe T, Pereon Y, Cauquil C, Colnaghi S, Mallucci G, Curro R, Tomaselli PJ, Thomas-Black G, Sullivan R, Efthymiou S, Rossor AM, Laura M, Pipis M, Horga A, Polke J, Kaski D, Horvath R, Chinnery PF, Marques W, Tassorelli C, Devigili G, Leonardis L, Wood NW, Bronstein A, Giunti P, Zuchner S, Stojkovic T, Laing N, Roxburgh RH, Houlden H, Reilly MM (2020). Cerebellar ataxia, neuropathy, vestibular areflexia syndrome due to *RFC1* repeat expansion. Brain.

[7] Infante J, Garcia A, Serrano-Cardenas KM, Gonzalez-Aguado R, Gazulla J, de Lucas EM, Berciano J (2018). Cerebellar ataxia, neuropathy, vestibular areflexia syndrome (CANVAS) with chronic cough and preserved muscle stretch reflexes: evidence for selective sparing of afferent Ia fibres. J Neurol.

[8] Cortese A, Simone R, Sullivan R, Vandrovcova J, Tariq H, Yau WY, Humphrey J, Jaunmuktane Z, Sivakumar P, Polke J, Ilyas M, Tribollet E, Tomaselli PJ, Devigili G, Callegari I, Versino M, Salpietro V, Efthymiou S, Kaski D, Wood NW, Andrade NS, Buglo E, Rebelo A, Rossor AM, Bronstein A, Fratta P, Marques WJ, Zuchner S, Reilly MM, Houlden H (2019). Biallelic expansion of an intronic repeat in *RFC1* is a common cause of late-onset ataxia. Nat Genet.

[9] Szmulewicz DJ, Roberts L, McLean CA, Macdougall HG, Halmagyi GM, Storey E (2016). Proposed diagnostic criteria for cerebellar ataxia with neuropathy and vestibular areflexia syndrome (CANVAS). Neurol Clin Pract.

[10] Sullivan R, Yau WY, Chelban V, Rossi S, O’Connor E, Wood NW, Cortese A, Houlden H (2020). RFC1 intronic repeat expansions absent in pathologically confirmed multiple systems atrophy. Mov Disord.

[11] Fan Y, Zhang S, Yang J, Mao CY, Yang ZH, Hu ZW, Wang YL, Liu YT, Liu H, Yuan YP, Shi CH, Xu YM (2020). No biallelic intronic AAGGG repeat expansion in *RFC1* was found in patients with late-onset ataxia and MSA. Parkinsonism Relat Disord.

[12] Akcimen F, Ross JP, Bourassa CV, Liao C, Rochefort D, Gama MTD, Dicaire MJ, Barsottini OG, Brais B, Pedroso JL, Dion PA, Rouleau GA (2019). Investigation of the *RFC1* repeat expansion in a Canadian and a Brazilian ataxia cohort: identification of novel conformations. Front Genet.

[13] Rafehi H, Szmulewicz DJ, Bennett MF, Sobreira NLM, Pope K, Smith KR, Gillies G, Diakumis P, Dolzhenko E, Eberle MA, Barcina MG, Breen DP, Chancellor AM, Cremer PD, Delatycki MB, Fogel BL, Hackett A, Halmagyi GM, Kapetanovic S, Lang A, Mossman S, Mu W, Patrikios P, Perlman SL, Rosemergy I, Storey E, Watson SRD, Wilson MA, Zee DS, Valle D, Amor DJ, Bahlo M, Lockhart PJ (2019). Bioinformatics-based identification of expanded repeats: a non-reference intronic pentamer expansion in *RFC1* causes CANVAS. Am J Hum Genet.

[14] Taki M, Nakamura T, Matsuura H, Hasegawa T, Sakaguchi H, Morita K, Ishii R, Mizuta I, Kasai T, Mizuno T, Hirano S (2018). Cerebellar ataxia with neuropathy and vestibular areflexia syndrome (CANVAS). Auris Nasus Larynx.

[15] Maruta K, Aoki M, Sonoda Y (2019). Cerebellar ataxia with neuropathy and vestibular areflexia syndrome (CANVAS): a case report. Rinsho Shinkeigaku.

[16] Nakamura H, Doi H, Mitsuhashi S, Miyatake S, Katoh K, Frith MC, Asano T, Kudo Y, Ikeda T, Kubota S, Kunii M, Kitazawa Y, Tada M, Okamoto M, Joki H, Takeuchi H, Matsumoto N, Tanaka F (2020) Long-read sequencing identifies the pathogenic nucleotide repeat expansion in *RFC1* in a Japanese case of CANVAS. J Hum Genet 10.1038/s10038-020-0733-y 32066831

[17] Wu TY, Taylor JM, Kilfoyle DH, Smith AD, McGuinness BJ, Simpson MP, Walker EB, Bergin PS, Cleland JC, Hutchinson DO, Anderson NE, Snow BJ, Anderson TJ, Paermentier LA, Cutfield NJ, Chancellor AM, Mossman SS, Roxburgh RH (2014). Autonomic dysfunction is a major feature of cerebellar ataxia, neuropathy, vestibular areflexia 'CANVAS' syndrome. Brain.

[18] Szmulewicz DJ, Merchant SN, Halmagyi GM (2011). Cerebellar ataxia with neuropathy and bilateral vestibular areflexia syndrome: a histopathologic case report. Otol Neurotol.

[19] Szmulewicz DJ, Waterston JA, Macdougall HG, Mossman S, Chancellor AM, McLean CA, Merchant S, Patrikios P, Halmagyi GM, Storey E (2011). Cerebellar ataxia, neuropathy, vestibular areflexia syndrome (CANVAS): a review of the clinical features and video-oculographic diagnosis. Ann N Y Acad Sci.

[20] Dolzhenko E, van Vugt J, Shaw RJ, Bekritsky MA, van Blitterswijk M, Narzisi G, Ajay SS, Rajan V, Lajoie BR, Johnson NH, Kingsbury Z, Humphray SJ, Schellevis RD, Brands WJ, Baker M, Rademakers R, Kooyman M, Tazelaar GHP, van Es MA, McLaughlin R, Sproviero W, Shatunov A, Jones A, Al Khleifat A, Pittman A, Morgan S, Hardiman O, Al-Chalabi A, Shaw C, Smith B, Neo EJ, Morrison K, Shaw PJ, Reeves C, Winterkorn L, Wexler NS, Group US-VCR, Housman DE, NG CW, Li AL, Taft RJ, van den Berg LH, Bentley DR, Veldink JH, Eberle MA (2017). Detection of long repeat expansions from PCR-free whole-genome sequence data. Genome Res.

[21] Shakkottai V, Paulson H (2019). Expanding the genetic basis of ataxia. Nat Genet.

[22] Delatycki MB, Corben LA (2012). Clinical features of Friedreich ataxia. J Child Neurol.

[23] Palma JA, Norcliffe-Kaufmann L, Kaufmann H (2018). Diagnosis of multiple system atrophy. Auton Neurosci.

[24] Kuzdas-Wood D, Irschick R, Theurl M, Malsch P, Mair N, Mantinger C, Wanschitz J, Klimaschewski L, Poewe W, Stefanova N, Wenning GK (2015). Involvement of peripheral nerves in the transgenic PLP-alpha-Syn model of multiple system atrophy: extending the phenotype. PLoS ONE.

[25] Sullivan R, Yau WY, O’Connor E, Houlden H (2019). Spinocerebellar ataxia: an update. J Neurol.

